# The role of placental hormones and metabolites in modulating uterine circulation in physiological and pathological pregnancies

**DOI:** 10.3389/fendo.2025.1637570

**Published:** 2025-08-19

**Authors:** Milena Esposito, Luana Paulesu, Maurizio Mandalà

**Affiliations:** ^1^ Department of Biology, Ecology & Earth Sciences, University of Calabria, Rende, Italy; ^2^ Department of Life Sciences, University of Siena, Siena, Italy; ^3^ Department of Obstetrics, Gynecology and Reproductive Sciences, University of Vermont Larner College of Medicine, Burlington, VT, United States

**Keywords:** uterine vasculature, feto-maternal interface, placental hormones, pregnancy complications, angiogenic factors

## Abstract

The adaptation of the uterine circulation during pregnancy is fundamental to ensure an adequate supply of oxygen and nutrients to the fetus, and this process is largely orchestrated by placental hormones/metabolites. In this review, we comprehensively examine the role of placental hormones, growth factors, and proteins in mediating vascular remodeling, vasodilation, and angiogenesis within the uterine circulation under both physiological and pathological conditions. Key molecules such as estrogens, progesterone, relaxin, VEGF, PlGF, and PTHrP, among others, promote structural and functional adaptations of uterine arteries, reduce vascular resistance, and enhance uteroplacental blood flow. Additionally, we discuss the impact of placental dysfunction on the development of pregnancy-related disorders such as preeclampsia, intrauterine growth restriction, gestational diabetes mellitus, and placenta accreta spectrum conditions that share common features of impaired uterine vascular remodeling and altered placental secretome. Furthermore, we explore innovative therapeutic strategies that aim to restore placental and vascular function, including gene therapy, mesenchymal stem cell-based approaches, and targeted nanomedicine. Finally, we highlight the emerging role of placental biomarkers for early diagnosis and risk stratification of vascular complications in pregnancy. Understanding the intricate interplay between placental secretions and the maternal vasculature is critical to advancing the prevention, diagnosis, and treatment of pregnancy complications, ultimately improving maternal and fetal health outcomes.

## 1 Introduction

Pregnancy requires the adaptation of the maternal uterine vascular system and the development of the placenta to establish the feto-maternal circulation. Within the maternal vascular system, the most prominent adaptation is the remodeling of the spiral uterine arteries (UAs) by trophoblast cells invasion to initiate the placentation process (1). As a result, the spiral UAs enlarge their opening diameters by 5- to 10-fold compared to the non-pregnant state and are transformed into low-resistance conduits that deliver blood at low pressure to the intervillous space (2). The changes in UAs structure include expansion of volume and the outgrowth of newly formed vessels, which are necessary to expand the vascular surface area and create a suitable environment for fetal growth. These morphological adaptations of the uterine circulation are accompanied by physiological changes in uterine vascular reactivity. Indeed, pregnancy is characterized by enhanced vasodilation and a blunted vasoconstrictive response to accommodate the increased utero-placental blood flow (UPBF). These adaptations are accomplished through several mechanisms, including the proliferative and vasodilatory effects of increased levels of circulating growth factors, as well as the effects of pro- and anti-inflammatory cytokines and hormones secreted by the placenta into the maternal bloodstream. Studies have demonstrated the involvement of these placental hormones and metabolites in regulating uterine circulation through pathways specific to endothelium and vascular smooth muscle cells (VSMCs). Alterations in endothelial nitric oxide synthase (eNOS) expression and activity, nitric oxide production (NO), and expression of enzymes involved in prostacyclin (PGI_2_) production contribute to the uterine artery endothelium-specific responses mediated by these bioactive molecules.

Therefore, the hormones and metabolites secreted by the placenta are crucial for the adaptations of uterine circulation, and hence for the development and survival of the fetus.

The placenta is a temporary endocrine organ that develops immediately after implantation during pregnancy. The placenta becomes fully functional by the 11^th^ week of gestation, and it regulates the bidirectional exchange of nutrients, oxygen and waste products between the fetal and maternal circulation. Despite accounting for less than 1% of maternal body weight, the placenta consumes approximately 40% of the oxygen supplied by the uterus at term (3), providing evidence for its high biosynthetic activity. The placenta secretes into the maternal circulation several hormones and metabolites, including estrogens, progesterone and relaxin, which are critical for the remodeling and the vasodilation of the uterine vasculature necessary to sustain the 40-fold increase in UPBF.

Therefore, placental insufficiency compromises feto-maternal circulation and has been frequently associated with pregnancy complications including preeclampsia (PE), intrauterine growth restriction (IUGR), gestational diabetes mellitus (GDM) and placenta accreta spectrum (PAS) disorders. These complications not only affect pregnancy outcomes but also have long-term consequences on offspring health. Thus, there is a need to maintain tight regulation of hormones/metabolites secreted by the placenta during pregnancy.

Throughout this review, the role of the placenta in mediating uterine vascular adaptations to pregnancy will be discussed in normal and complicated pregnancies such as PE, IUGR, GDM and PAS. Thus, understanding how hormones and proteins secreted by the placenta affect the uterine circulation in both physiological and pathological pregnancies is of interest for improving maternal and fetal outcomes.

## 2 Physiological regulation of uterine circulation by placental hormones and growth factors

**Figure 1:**
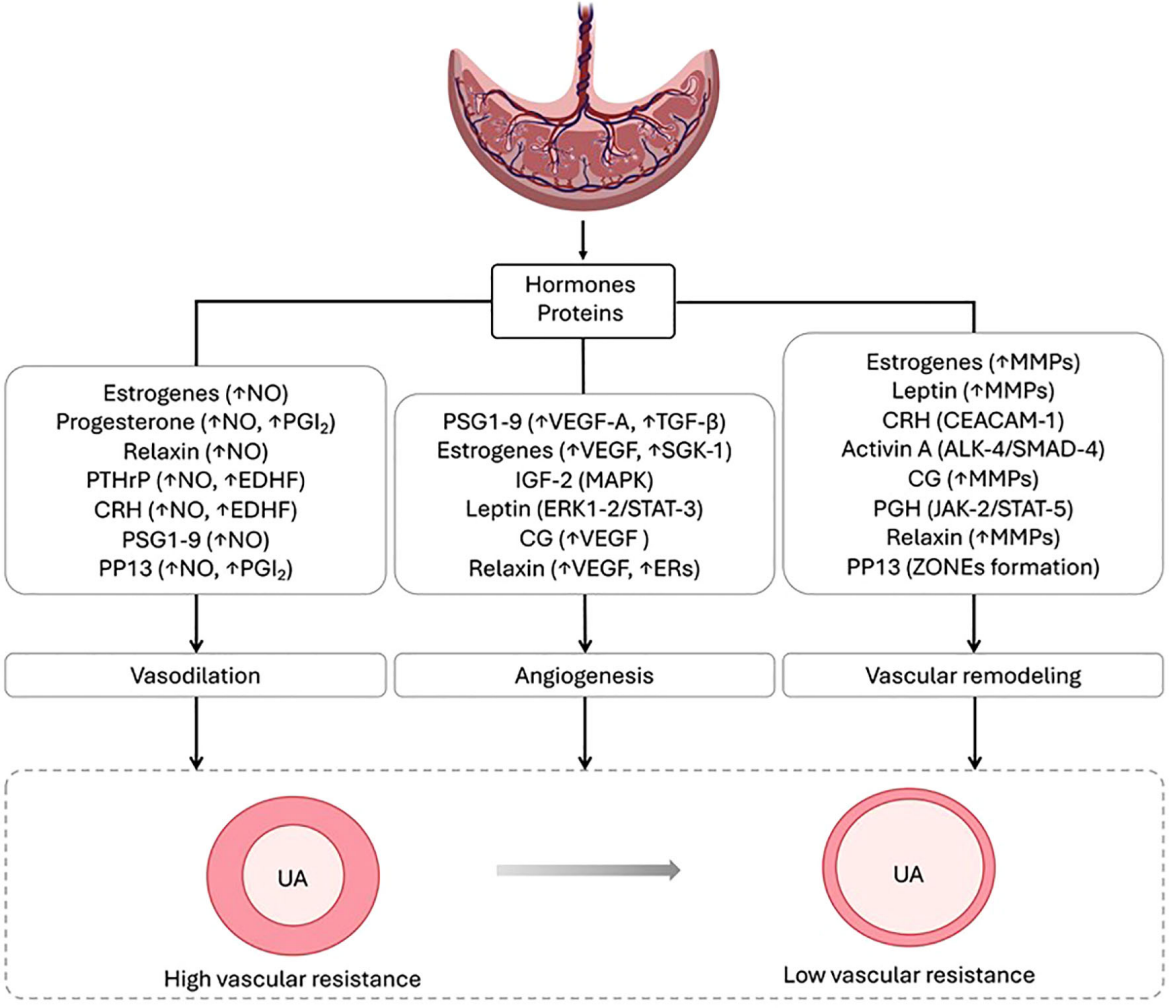
Mechanisms by which placental factors modulate uterine vascular adaptations to pregnancy. The figure illustrates the main placental factors and signaling pathways mediating vasodilation, angiogenesis and uterine vascular remodeling during pregnancy. The coordinated actions, transforming the UA into dilated vessel with low vascular resistance, thus sustaining the increase in UPBF and supporting a proper fetal growth.

The placenta exhibits massive endocrine activity. The coordinated action of hormones, proteins and growth factors synthesized by the placenta is essential in regulating UA expansion and reducing vascular resistance, supporting the increased UPBF required for fetal development (**Figure 1**).

The following section summarizes studies conducted on animals, humans, as well as cell culture, evaluating the role of hormones, growth factors and protein synthesized by the placenta, to elucidate their effects and mechanisms of action in processes underlying uterine vascular remodeling during pregnancy (**Table 1**).

**Table 1:** Effects of placental factors in mediating vascular adaptations to pregnancy.

Molecules	Samples	Species	Effects	Reference
Hormones				
E_2_β and metabolites	UAECs	ewe	angiogenesis	(6)
	MUA	rat	vasodilation	(13)
	UAECs	ewe	vasodilation?	(294)
	UA	ewe	vasodilation?	(17)
	UA	rat	vasodilation	(14)
	CTBs	human	vascular remodeling	(9)
	Placental basal plate	baboon	vascular remodeling	(12)
	Uterus	rat	vascular remodeling	(11)
Estrogenic agonist	Placental artery; Myometrial UA	human	vasodilation	(15)
	RUA	rat	vasodilation	(16)
Progesterone	UA	ovine	vasodilation	(25)
	Uterus	rat	vascular remodeling	(11)
	UA	ovine	vasodilation	(27)
	UA	ovine	vasodilation	(28)
Relaxin	UA; myometrium	rat	vasodilation	(35)
	SMC	mice	vascular remodeling	(32)
	UA	rat	vascular remodeling	(34)
	UA	mice	vascular remodeling	(33)
	Endometrial stromal cells	human	angiogenesis; vascular remodeling	(30)
PTHrP	UA	rat	vasodilation	(66)
	Placental artery	human	vasodilation	(68)
	Placental artery	human	vasodilation	(67)
IGF-2	UMVECs	human	angiogenesis	(73)
PL	MUA	goats	↔ UBF	(91)
	Serum/morphometric analysis	ewe	↓UBF?	(93)
	CAM membrane; BBCE cells		angiogenesis	(94)
leptin	HTR8/SVneo; CBTs; chorionic villous explants	human	vascular remodeling	(103)
	CTBs	rat	vascular remodeling	(104)
	CTBs	human	vascular remodeling	(105)
	UAECs	ewe	proliferation (angiogenesis)	(106)
	UVECs; aortic ECs	human, porcine	angiogenesis	(107)
CRH	Placental villi	human	vasodilation	(110)
	UA	rat	vasodilation	(111)
	EVTs	human	vascular remodeling	(112)
NPY	UA	guinea pig	↓ vasoconstriction	(114)
	placenta	human	angiogenesis? vasodilation?	(117)
Activin A	HTR8/SVneo; EVTs	human	vascular remodeling	(123)
	HTR8/SVneo;EVTs; chorionic villous explants	human	vascular remodeling	(124)
	HTR8/SVneo;EVTs; chorionic villous explants	human	vascular remodeling angiogenesis	(125)
	HTR8/SVneo	human	vascular remodeling	(126)
PSG- 1/9	UVECs; C57BL/6	Human; mice	vascular remodeling vasodilation ()?	(130)
	UVECs	human	vasodilation	(131)
	HTR-8/SVneo	human	angiogenesis	(132)
PGH	EVCs	human	vascular remodeling	(95)
Proteins				
CG	UMVECs	human	angiogenesis	(80)
	CTBs	human	vascular remodeling	(82)
	Corpus luteum	human	angiogenesis	(81)
	RUA	rat	vasodilation	(83)
PP13	UA	human	vasodilation	(90)
	UV	rat	vasodilation	(89)
	UAA	rat	vasodilation	(87)
	UV	rat	vascular remodeling	(86)
	Endometrial tissue	human	vascular remodeling	(85)

AC, adenylyl cyclase; AKT, protein kinase B; ALK-4, activin receptor-like kinase 4; BBCE, bovine brain capillary endothelial cells; BKca, big conductance calcium-activated potassium channel; Ca^2+^, calcium ion; CAM membrane, chorioallantoic membrane; CEACAM-1, carcinoembryonic antigen-related cell adhesion molecule 1; cGMP, cyclic guanosine monophosphate; COX-1, -2, cyclooxygenase-1 and -2; CRHR-1, corticotropin-releasing hormone receptor 1; CTBs, cytotrophoblasts; ECs, endothelial cells; EDHF, endothelium-derived hyperpolarizing factor; eNOS, endothelial nitric oxide synthase; ER-α, -β, estrogen receptor alpha and beta; ERK-1, -2, extracellular signal-regulated kinases 1 and 2; EVTs, extravillous trophoblasts; HTR8/SVneo, human trophoblast cell line 8/SV40 large T antigen; IGF-2, insulin-like growth factor 2; JAK-2, Janus kinase 2; K_v_, voltage-gated potassium channels; LH-R, luteinizing hormone receptor; M6PR, mannose-6-phosphate receptor; MAPK, mitogen-activated protein kinase; MMP-2, matrix metalloproteinase-2; MMPs, matrix metalloproteinases; MUA, main uterine artery; NO, nitric oxide; PGH-R, prostaglandin H receptor; phospho-STAT3, phosphorylated signal transducer and activator of transcription 3; PI3K, phosphoinositide 3-kinase; cPLA_2_, phospholipase A_2_; PGIS, prostacyclin synthetase; PKC, protein kinase C; RUAs, radial uterine arteries; RXFP-1, relaxin family peptide receptor 1; sGC, soluble guanylyl cyclase; SGK-1, serum and glucocorticoid-regulated kinase 1; SKca, small conductance calcium-activated potassium channel; SMAD, small mothers against decapentaplegic; SMC, smooth muscle cell; Stat-5, signal transducer and activator of transcription 5; TGFB-1, transforming growth factor beta 1; UAA, uterine arcuate artery; UAECs, uterine artery endothelial cells; UA, uterine artery; UMVECs, umbilical microvascular vein endothelial cells; UV, umbilical vein; VEGF, vascular endothelial growth factor; Y2R, neuropeptide Y receptor 2; ZONEs, zones of necrosis. ↑ = increase; ↓ = decrease; ↔ = no effect;? = indirect effect.

### 2.1 Estrogens

The placenta becomes the primary source of estrogen after week 9 of human pregnancy, when levels of estradiol increase by 50-fold during pregnancy (4). *In vitro* studies have demonstrated that estradiol-17β induces proliferation, migration and adhesion of endothelial cells (ECs) (5–8), critical processes underlying uterine angiogenesis and facilitating the increase in UPBF for a healthy pregnancy establishment. These effects have been observed in both human umbilical vein endothelial cells (HUVECs) and uterine arterial endothelial cells (UAECs) (5–8). In addition, estrogens contribute to uterine vascular remodeling by enhancing the transcriptional activity of serum and glucocorticoid-inducible kinase-1, which in turn modulates matrix metalloproteinases-2 (MMP-2) and E-cadherin expression in human villous samples (9).

Animal studies further demonstrate the role of estrogens in promoting vascular remodeling and hemodynamic adaptation. During early primate pregnancy, estrogens promote trophoblast cell proliferation, differentiation, viability, and invasion of the spiral UA (10). In pregnant rats, estradiol was shown to activate extracellular MMPs inducer, which in turn increased the expression and activity of MMP-2 and MMP-9 in uterus and aorta of late-pregnant rats vs virgin and mid-pregnant rats, supporting its role in vascular remodeling (11). However, in baboons, as gestation advances, the rise of estrogenssuppresses trophoblast uterine invasion (10) by inhibiting vascular endothelial growth factor (VEGF) expression and increasing soluble fms-like tyrosine kinase-1(sFlt-1) levels (12).

Furthermore, several studies have shown that estrogens vasodilate UA (13–16), favoring the augmentation in UPBF during pregnancy. In pregnant ewes, systemic infusion of estradiol-17β induced a 5-fold increase in UPBF, mainly mediated by large-conductance Ca^2+^-activated K^+^ channel (BK_ca_) and NO (17). In rats, estrogens were shown to act through the G protein-coupled estrogen receptor (GPER) (18, 19), promoting endothelium-dependent vasodilation via the NO–cyclic guanosine monophosphate (NO–cGMP) signaling pathway (20). GPER-mediated vasodilation also involves L-type Ca^2+^ channels and ERK1/2 activation in VSMCs (16).

In human myometrial arteries, vasodilation in response to 17β-estradiol is mediated by the greater expression of estrogen receptors-α, and -β (ER-α, -β) (15).

The effects of estrogens on uterine circulation during pregnancy have been extensively reviewed elsewhere (18, 21).

### 2.2 Progesterone

Progesterone is a steroid hormone whose levels during pregnancy are almost 10 times higher than during the luteal phase of the menstrual cycle (22). Around the 10^th^ week of gestation, the placenta takes over progesterone production from the corpus luteum, maintaining serum concentrations of at least 10 ng/ml, which are essential for sustaining a healthy pregnancy (23). Progesterone contributes to thickening and expanding the endometrial lining, thereby enlarging the surface area available for implantation of the fertilized egg (24). It also facilitates trophoblastic invasion of the spiral UA, thereby promoting growth and remodeling of the uterine vasculature and increasing UPBF. Moreover, progesterone induces uterine vessel vasodilation by acting on both ECs and VSMCs. In ECs, it upregulates the expression and activity of eNOS (25) as well as cyclooxygenase-1 and -2 (COX-1, COX-2) (26), increasing the production of NO and PGI_2_ respectively (27). In VSMCs, progesterone downregulates the protein kinase C pathway (PKC), further contributing to vessel vasodilation (28).

Furthermore, progesterone, along with estrogen, promotes vascular remodeling by enhancing MMPs expression and activity (11). However, the effect of progesterone in regulating UPBF during pregnancy appears controversial (25); therefore, further studies are needed to elucidate its effects and the molecular mechanisms through which progesterone modulates the uterine vascular function during pregnancy.

### 2.3 Relaxin

Relaxin is produced mainly by the corpus luteum and, during pregnancy, also by the placenta. Relaxin reaches a peak of about 1 ng/ml by the end of the first trimester, then its levels gradually decline and plateau at ~0.6 ng/ml (29). Relaxin contributes to the growth and softening of the cervix, facilitating rapid labor, and it also contributes to uterine vasodilation and increases compliance, favoring the augmentation in UPBF (29). *In vitro* studies have demonstrated that relaxin stimulates angiogenesis. Experiments on human endometrial stromal cells showed that relaxin treatment induces a variety of angiogenesis-related genes, including VEGF-A (30). Moreover, relaxin has been shown to induce VEGF and basic fibroblast growth factor (31) expression in ischemic wound sites, supporting its pro-angiogenic activity.

Animal studies have provided strong evidence for relaxin role in vascular remodeling and uterine compliance. Kaftanovskaya et al., have demonstrated that relaxin-receptor-deficient mice had reduced lengths of the pubic symphysis and increased collagen density in reproductive tissues (32). Similarly, in pregnant relaxin-mutant mice, UA exhibited increased elastin expression and decreased levels of MMPs and adhesion molecules. Interestingly, 5 days of exogenous relaxin treatment reversed arterial stiffness and improved fetal weight in relaxin-deficient mice (33). Consistent with these findings, neutralization of relaxin in late pregnancy using the monoclonal antibody-1 (MCA-1) has been shown to increase uterine artery stiffness (34). Moreover, relaxin induced vasodilation in UA isolated from pregnant rats, with a greater effect during mid- compared to late pregnancy through the NO-sGC pathway (35). Recent evidence suggests that the vasodilatory responses of relaxin are mediated by its major receptor, the relaxin/insulin-like family peptide 1 receptor (RXFP1), which is most highly expressed in the UA during early pregnancy (34). Newly emerging data support that relaxin binds to RXFP1 activates Gαi/o protein, coupling to phosphatidylinositol-3 kinase/Akt (protein kinase B)-dependent phosphorylation and activation of eNOS (36). Nonetheless, relaxin inhibits spontaneous myometrial contractile activity in mid-gestation, while it has no effect at term (35).

Human studies suggest a potential role of relaxin in regulating uteroplacental vascular function. A positive correlation between serum relaxin levels and UA resistance index at 10–12 weeks of gestation has been observed, suggesting that relaxin contributes to the regulation of the uteroplacental vasculature. Moreover, suppression of circulating relaxin throughout mid-pregnancy abolished the cardiovascular adaptations required to sustain a healthy pregnancy (37). Thus, the sensitivity of the uterus to relaxin is subjected to modulation through pregnancy, suggesting that relaxin sustains an adequate UPBF during mid-gestation, while facilitating labor at term, allowing for vasoconstriction.

In summary, while animal studies show promising roles in vascular remodeling and compliance, human studies are limited and mostly observational, lacking mechanistic insights into RXFP1 signaling in the uterine vasculature during normal and pathological pregnancies

### 2.4 Vascular endothelial growth factor

In addition to steroid hormones, the placenta secretes a wide range of angiogenic factors, including VEGF, placental growth factor (PlGF) and sFlt-1, which collectively contribute to the regulation of the uterine circulation. In healthy pregnancies, maternal plasma VEGF concentrations are markedly elevated compared to non-pregnant baselines. It rises during the first trimester, peaks at 10–14 weeks, remains elevated up to the 20^th^ week of pregnancy, then declines in the third trimester (38, 39). During the first trimester of pregnancy, VEGF levels increase 4–5 fold (163.2 ± 81.6 pg/mL) compared to the non-pregnant state (18.5 ± 16.8 pg/mL) (40). VEGF contributes to maternal adaptations to pregnancy by increasing vascular permeability, stimulating angiogenesis and inducing vasodilation of the uterine circulation.

The expression of VEGF mRNA in both the uterine endometrium (41) and placenta (42) indicates its active role at the feto-maternal interface, where tightly regulated modulation of vascular permeability and angiogenesis is crucial for the establishment of a successful uteroplacental circulation. VEGF-mediated vascular permeability has been demonstrated in the uterine vasculature and is further enhanced by pregnancy (43, 44). The increase in permeability facilitates the extravasation of plasma proteins into surrounding tissue, facilitating ECs migration and thus the angiogenic process (45). In addition, VEGF induces the expression of serine proteases urokinase, tissue-type plasminogen activators, PA inhibitor 1 (46) and MMPs interstitial collagenase (47), consistent with a pro-degradative environment, which facilitates migration and sprouting of ECs during angiogenesis. Notably, VEGF angiogenic properties are enhanced during pregnancy (48). VEGF role in mediating vasodilation has also been shown *in vitro*. Indeed, VEGF promotes NO production in cultured UAECs (48) and stimulates PGI_2_ synthesis in a time- and concentration-dependent manner in UVECs via activation of cytosolic phospholipase A_2_/p42/p44 MAP kinases pathways (49).

The critical role of VEGF in angiogenesis is supported by evidence from genetically modified pregnant mice lacking VEGF gene or deficient in VEGF receptors, both of which result in impaired vascular development and pregnancy loss (50, 51). VEGF promotes NO production *in vivo*, in the UA of pregnant ovine (52), while reducing UA contractility and increasing UPBF (52–54) short- and long-term. Consistent with these findings, VEGF induced vasodilation of uterine arcuate artery (UAA) of both non-pregnant and pregnant rats, acting through an endothelium-dependent mechanism (55).

### 2.5 Placental growth factors

PlGF belongs to the VEGF growth factor family, and it binds specifically to VEGFR-1 (56). Plasma PlGF concentrations increase from week 11 to 12 onward to peak at week 30 in healthy pregnancy (57), coinciding with implantation and early vascular development. PlGF is expressed in villous trophoblasts and vascular endothelium in human placenta at term (58), and the correlation between PlGF expression and placental perfusion suggests that PlGF may contribute to ensuring adequate vascular development and function of the placenta early in gestation (59). PlGF contributes to uteroplacental circulation establishment by supporting angiogenesis, immune modulation and trophoblast invasion. Its role in vascular remodeling is supported by a study demonstrating a reduced uterine natural killer population in PlGF null mice (60). This sub-population of natural killer cells is the major participant in the early vascular changes in the pregnant endometrium. Thus, demonstrating that PlGF-mediated remodeling of the UA at the feto-maternal interface is necessary to sustain an adequate UPBF. Supporting this finding, an increased level of PlGF correlates with improved placental perfusion (59). In addition, PlGF potentiates the angiogenic response to VEGF on microvascular ECs (61), thereby promoting the angiogenic process at the feto-maternal interface. PlGF regulates the UPBF, inducing vasodilation, as demonstrated in placental arteries (62) and uterine circulation of rats and humans (63). The mechanism of PlGF-mediated vasodilation has been associated with signaling through VEGFR-1 and NO involvement, according to the vascular bed (63).

### 2.6 Additional placental hormones and proteins involved in vasodilation, vascular remodeling and angiogenesis of the uterine circulation during pregnancy

In addition to the well-known sex hormones and growth factors discussed above, whose role in pregnancy-associated uterine vascular remodeling has been widely demonstrated and established, several studies have suggested other placental hormones, proteins and growth factors as critical contributors to a successful uterine vascular remodeling.

#### 2.6.1 Parathyroid hormone-related protein

PTHrP is expressed in the placenta and fetal tissues, and its expression increases as gestation progresses (64, 65). It plays a crucial role in placental calcium transport, a function important in maintaining fetal calcium homeostasis. Furthermore, PTHrP regulates uterine vascular tone during pregnancy, inducing vasodilation in the UA of both non-pregnant and pregnant rats (66) and human feto-placental vasculature (67, 68). This vasodilatory effect is mediated through the PTH1 receptor (PTH1R) and involves NO and prostacyclin production, leading to relaxation of VSMCs (66, 69). PTHrP increases the expression of COX-2 and the production of 8-iso-prostaglandin F2α, which in turn can regulate the expression of PTH1R, suggesting a feedback mechanism in VSMCs (70). Alterations in PTHrP expression have been associated with pregnancy complications. For instance, decreased levels of PTHrP have been observed in the placentas of spontaneously hypertensive rats, and they have been shown to correlate with IUGR (71). In conclusion, PTHrP is a multifunctional protein that, through its vasodilatory effects, regulation of vascular tone, and facilitation of calcium transport, ensures adequate UPBF and supports fetal development. However, despite these findings, the precise molecular mechanisms by which PTHrP regulates uterine vascular remodeling remain poorly defined, further human studies evaluating PTHrP levels in pregnancy complication are still lacking. Future research should also clarify whether PTHrP modulation could offer therapeutic benefits in pregnancy disorders.

#### 2.6.2 Insulin growth factor

Insulin growth factor 2 (IGF-2) is abundantly expressed in trophoblast cells and fetal tissue (72). Studies on human uterine microvascular endothelial cells (UMVECs) have demonstrated that IGF-2 promotes ECs migration through the mannose 6-phosphate receptor (73). In addition, IGF-2 has been shown to promote endothelial proliferation and cell survival in placental villous explants, indicating a broader function in placental angiogenesis. The importance of IGF-2 in fetal development is further underscored by studies in mice, where IGF-2 deficiency resulted in growth restriction (74), indirectly supporting its action on UPBF. Thus, IGF-2 contributes to the vascular adaptation to pregnancy mainly by promoting angiogenesis.

Although the role of IGF-2 in placental angiogenesis and fetal growth has been documented gaps remain in understanding its effects on the maternal uterine circulation in the context of vascular tone and UPBF regulation. Further, the molecular pathways through which IGF-2 might influence NO production or expression, as well as PGI_2_ are largely unexplored. Future studies should clarify these mechanisms particularly through *in vivo* models and functional assessment of uterine circulation to better define IGF-2 contribution to maternal vascular adaptation during pregnancy.

#### 2.6.3 Pregnancy-associated plasma protein A

PAPP-A is a proteinase highly expressed during pregnancy (75). PAPP-A enhances IGF bioavailability (76), which in turn promotes trophoblast invasion and angiogenesis, leading to a proper vascular remodeling required for adequate uteroplacental perfusion and fetal development. Low maternal serum PAPP-A has been associated with high UA resistance index (77), suggesting PAPP-A involvement in the regulation of UBPF.

Although PAPP-A is associated with improved IGF bioavailability and favorable pregnancy outcomes, come mainly from observational rather than direct mechanistic findings. The mechanisms by which PAPP-A modulates UA vasodilation and vasoconstriction events during pregnancy, as well as the downstream effects of altered PAPP-A expression on uterine microvascular networks beyond the spiral UA remain poorly defined.

#### 2.6.4 Human chorionic gonadotropin

hCG is a glycoprotein hormone produced predominantly by placental syncytiotrophoblast. hCG receptors have been identified on uterine ECs (78, 79), and hCG promotes angiogenesis by stimulating ECs migration and capillary sprouting (80). Evidence from luteal tissue also supports its pro-angiogenic properties, increasing proliferation of ECs and expansion of both endothelial and pericyte compartments, thus contributing to vascular stabilization during pregnancy (81). Additionally, hCG increases migration, proliferation and invasion of trophoblast cells into the maternal decidua and the subsequent remodeling of spiral UA into low-resistance, high-capacitance vessels, essential for adequate placental perfusion (82). Moreover, hCG induces vasodilation of UA through the NO pathway, reducing vascular resistance and enhancing UPBF (83).

However, the downstream intracellular signaling pathways mediating hCG’s pro-angiogenic and vasodilatory actions—beyond NO—are not comprehensively characterized. Longitudinal studies examining hCG fluctuations and uterine hemodynamics throughout pregnancy, along with experimental models manipulating hCG levels, would help clarify its direct role in uterine vascular adaptation and disease susceptibility.

#### 2.6.5 Placental protein 13

PP13, also known as galectin-13, is a member of the galectin family predominantly secreted from placental syncytiotrophoblasts. In addition to its immunomodulatory function (84), PP13 contributes to the vascular remodeling of the spiral UA (85) and uterine veins (UV) (86), ensuring adequate UPBF to the fetus. *In vivo* studies have demonstrated that PP13 administration lowered blood pressure and promoted placental and fetal growth (86). These findings are further supported by ex vivo evidence demonstrating PP13 vasodilation effects in UA from both rats (87–89) and humans (90). The vasodilation action of PP13 is mediated, at least in part, through the NO pathway. By promoting increased UPBF, PP13 supports fetal development and contributes to the maintenance of a healthy pregnancy.

Although the contribution of PP13 in mediating the uterine vascular adaptation to pregnancy has been widely demonstrated in animal models or ex vivo, there is a lack of human *in vivo* data confirming its role in UPBF regulation.

#### 2.6.6 Placental lactogen and placental growth hormone

PL and GH-V are members of the growth hormone/prolactin family expressed in the human syncytiotrophoblast. Although a direct association between PL and UPBF has not been definitively established (91), maternal circulating levels of PL have been positively associated with fetal weight (92). Consistent studies in animal models have demonstrated that PL deficiency resulted in reduced fetal and placental weight (93), indirectly indicating a potential contribution of PL to adequate UPBF necessary for normal fetal growth. The full-length hormone PL stimulates blood vessel formation *in vivo* at different stages of CAM development (94), thus sustaining its potential pro-angiogenic effect at the feto-maternal interface. However, the role of PL in mediating the vascular adaptation to pregnancy remains inconclusive and needs to be further elucidated.

GH-V is specifically expressed in the syncytiotrophoblast and invasive EVTs of the human placenta (95). It has been shown to promote EVTs invasive-phenotype *in vitro* (95), which is an essential process for spiral UA remodeling. Further, its pro-angiogenic effect has been proved in bovine brain capillary cells (94). GH-V correlates with the increases in circulating IGF-1 observed during pregnancy (96) and it is associated with fetal growth (97, 98). However, conflicting results exist on the relationship between maternal GH-V/IGF-1 concentrations and fetal growth during pregnancy (98).

In summary, while PL promote angiogenesis, GH enhances EVT invasion and is associated with IGF-2 increases. However, direct evidence linking PL and GH with uterine hemodynamics in humans is missing. Further, data on receptors and downstream pathways in ECs or VSMCs needs to be clarified.

#### 2.6.7 Leptin

Leptin, a peptide hormone primarily secreted by adipocytes, is also produced by the placenta, particularly by syncytiotrophoblasts (99). Leptin treatment resolves the infertility in obese female mice (100) and it is essential for implantation processes (101). Leptin is likely to play a role in placental development, as its receptors have been identified on placental cytotrophoblasts (CTBs) (102). *In vitro* studies have demonstrated that leptin promotes CTB invasion (103) through STAT3, PI3K, MAPK pathways (103) and partly by modulating MMPs activity (104). Specifically, leptin increased the secretion of MMP-2 and fetal fibronectin and enhanced the activity of MMP-9 (105). Further, leptin treatment has been shown to induce angiogenesis in UAECs (106), in UVECs and in porcine aortic ECs (107). Although leptin has demonstrated vasodilation effects in the human forearm (108), its direct vasoactive role in the uterine circulation remains to be elucidated. Further, the balance between the pro-angiogenic vs pro-inflammatory effects leptin-mediated in the uterine vasculature is poorly understood, thus further studies need to be addressed.

#### 2.6.8 Corticotropin-releasing hormone

CRH is secreted by syncytiotrophoblast cells of the human placenta into both the fetal and maternal circulation (109). It regulates the hypothalamic-pituitary-adrenal axis and the initiation of labor. Further, CRH acts as a vasodilator of the feto-maternal circulation (110) and UA from pregnant rats via NO–cGMP-EDHF signaling (111). Hence, providing evidence that CRH may act as a regulator of the UPBF. Furthermore, CRH has been shown to attenuate the invasiveness of EVTs, thereby contributing to ensuring controlled remodeling of spiral UA (112). The dual action of promoting vasodilation and regulating trophoblast invasion demonstrates that CRH contributes to the remodeling of the spiral UA. Most of the vasodilatory evidence is from animal models; confirmation in human uterine arteries is limited, further, the interaction with other placental hormones in regulating vascular tone is poorly explored.

#### 2.6.9 Neuropeptide Y

NPY is widely expressed in the central and peripheral nervous systems, and it is also produced by placental and fetal membranes (112). NPY regulates UPBF mainly by inducing vasoconstriction through Y1 receptors located on VSMCs (113). However, UAs isolated from pregnant animals showed a reduced sensitivity to NPY (114), which suggests a finely tuned regulatory mechanism to balance vascular tone and ensure adequate UPBF. In normal placentas, Y2R- the NPY receptor involved in promoting angiogenesis (115) and vasodilation (116)- is the most abundantly expressed among the NPY receptors (117). These changes in receptor expression patterns support a role for NPY in the vascular adaptation of pregnancy. Nonetheless, the specific contribution of NPY and Y2R in regulating UA vascular tone and angiogenesis during pregnancy needs to be further investigated.

#### 2.6.10 Inhibin A

Inhibin A is produced by placental syncytiotrophoblast during pregnancy, and its circulating levels increase as pregnancy progresses. While direct evidence of inhibin A role in uterine vascular remodeling is limited, its elevated levels have been associated with PE and IUGR (118), suggesting a potential role of inhibin A in placental function and uterine perfusion during pregnancy. To the best of our knowledge, no direct evidence supports vasodilation or angiogenic properties of inhibin A on the uterine circulation during pregnancy, although inhibin A induces angiogenesis in pathological processes (119). Therefore, given the similarities between tumors and the human placenta, the pro-angiogenic properties of inhibin A on uterine circulation during gestation remain to be elucidated.

#### 2.6.11 Activin A

Activin A is a member of the transforming growth factor-beta (TGF-β) superfamily and the placenta is the major source of activin A throughout pregnancy (120). The importance of activin A in sustaining a physiologic pregnancy is suggested by the association between its levels and pregnancy complications (121). Activin A receptors are expressed on human trophoblast cells (122) and their role in promoting trophoblast cells invasion, differentiation and migration has been widely demonstrated in HTR8/SV neo cell culture (123–126). Further, Activin A has been shown to enhance VEGF expression, thereby promoting angiogenesis (126). Interestingly, in UVECs activin A promotes the expression of adhesion molecules (127), contributing to the maternal endothelial dysfunction observed in women with PE (128). Although direct evidence of a vasodilatory effect of activin A on UA is lacking, its involvement in trophoblast-endothelial crosstalk suggests a role in mediating the adaptations of the uterine circulation to pregnancy.

#### 2.6.12 Pregnancy-specific β-glycoproteins

PSGs are secreted mainly by trophoblast cells throughout gestation (129). During pregnancy, members of the PSGs family regulate the vascular function, promoting angiogenesis and vasodilation. *In vitro*, treatment with PSG-1 enriched exosomes promotes UVECs proliferation and migration, as well as NO release (130). Consistent with these findings, Qin et al., have demonstrated a role of PSG-9 in increasing NO production through the enhanced expression levels of store-operated calcium entry channels proteins (131). In addition, PSG-1 induces angiogenesis via TGFB-1 and VEGF-A, thus contributing to the establishment of the feto-maternal circulation during pregnancy (132). However, the direct effects of PSGs on uterine artery reactivity and spiral artery remodeling *in vivo* are largely unexplored. Moreover, the specific contributions of different PSG isoforms beyond PSG-1 and PSG-9 to uterine vascular adaptation remain unclear. Further research is needed to elucidate the receptor-mediated pathways involved and to investigate whether PSGs exert differential effects in normal versus pathological pregnancies.

## 3 Pathological conditions and dysregulation of uterine circulation

Appropriately timed pregnancy-dependent changes in vasculature are critical for healthy pregnancy outcomes. Thus, alterations in the secretion of placental hormones and metabolites compromise the physiological adaptation of the uterine circulation, leading to reduced UPBF. Most of the pregnancy losses occur early in gestation, when the placenta is established. These losses may be associated with aberrations in remodeling of the uterine vasculature and angiogenesis imbalance at the feto-maternal interface, resulting in pregnancy complications such as PE, IUGR, GDM, and PAS as discussed in the following section.

### 3.1 PE

PE is defined as new-onset hypertension after 20 weeks of pregnancy, with or without proteinuria and evidence of end-organ damage. Clinically, PE is associated with several maternal and fetal complications (133), and is generally classified in early-onset (<34 weeks of gestation), which tends to be more severe, and late-onset (>34 weeks of gestation), which is usually milder. The placenta has a crucial role in the pathophysiology of both early- and late-onset PE, though the specific mechanisms differ. Early-onset PE is more strongly associated with abnormal placentation, poor uterine perfusion, and IUGR (134), whereas late-onset is primarily attributed to predisposing maternal factors and placental senescence (134).

It is generally accepted that PE originates from the abnormal transformation of the spiral arteries underlying the placenta, due to impaired invasion of the uterine wall by migrating trophoblast cells (135, 136). In addition to incomplete or absent vascular remodeling, signs of vascular damage, resembling atherosclerosis-like lesions, have been described in placental bed samples from women with PE compared to women with healthy pregnancies (135). This maladaptive phenotype leads to narrower maternal uterine vessels and relative placental ischemia, ultimately reducing the UPBF as demonstrated by Doppler ultrasound (137–139).

Numerous hormones and proteins of placental origin have been investigated to elucidate the molecular mechanisms underlying the aberrant uterine vascular phenotype observed in PE. It is well-established that PE is associated with a decrease in pro-angiogenic factors such as PlGF and VEGF, coupled with an increase in anti-angiogenic factors such as sFlt-1 and sEng.

Supporting this notion, a reduction in circulating PlGF has been associated with placental hypoperfusion in both human (140) and animal models of PE (141). Placental hypoperfusion, indicative of defective adaptations of the uterine circulation, may be partially explained by a substantial elevation of sFlt-1 observed in PlGf^-/-^ mice (142). Notably, PlGF administration dampened sFlt-1 increase, restoring angiogenic signaling in a PE-like model (141). The anti-angiogenic effects of sFlt-1 are further exacerbated by sEng, which has been shown to induce a severe state of PE in pregnant rats (143). The exacerbation of the PE-like symptoms is further supported by *in vitro* evidence showing that, in UVEC, sEng treatment contributes to endothelial dysfunction by inhibiting NO production, reducing cell viability, impairing trophoblast invasiveness, as well as suppressing MMPs expression (144).

Thus, PE is characterized by a shift toward an antiangiogenic profile, which disrupts the normal vascular homeostasis, resulting in endothelial cell dysfunction, including decreased NO production, and release of procoagulant proteins. Hence, contributing to hypertension and reduced UPBF.

### 3.2 IUGR

IUGR is defined as fetal weight estimated to be below the 10^th^ percentile for its gestational age. The most common cause of IUGR is poor placental function and placental ischemia due to deteriorated uteroplacental perfusion (145, 146). A strong association between IUGR and PE has been consistently observed, with IUGR frequently arising in pregnancies complicated by severe PE (147). Histopathological observations revealed a significant decrease in villous vascular density (148) and a maldevelopment of the placental terminal villous tree in placentae from IUGR pregnancies compared to those from normal pregnancies (149). These findings suggest that the aberrant vascular formation is a leading determinant in IUGR, explaining the sub-optimal UPBF. From a molecular standpoint, the angiogenic profile of IUGR placentas presents inconsistencies that may reflect compensatory and pathogenic mechanisms. A study from Barut et al., reported increased placental expression of VEGF-A, basic fibroblast growth factor and eNOS, in placental samples from IUGR mothers compared to control samples collected during the third trimester (150). This result suggests a possibly compensatory upregulation aimed at resolving placental hypoxia. However, Lyall et al. (151), found a reduction in VEGF expression in placental villous tissue from pregnancies complicated by IUGR and PE, indicating that angiogenic insufficiency may dominate in such pathological contexts. Similarly, the expression of PlGF mRNA in placental samples from IUGR pregnancies has been inconsistent, with evidence demonstrating a decrease in PlGF expression only in severe cases of IUGR (152). Study using an ovine model of IUGR has demonstrated an increase in VEGF mRNA levels in the IUGR group compared to the control in early gestation, possibly reflecting early compensatory mechanisms. However, during mid-gestation, the expression of VEGF receptors in fetal tissue was significantly reduced, potentially limiting VEGF and PlGF signaling, thereby impairing angiogenesis and placental vascular development (153).

This finding may reflect a progressive failure of the placenta to sustain proangiogenic signaling under chronic stress, hence affecting UPBF.

### 3.3 Gestational diabetes mellitus

GDM, defined as hyperglycemia first diagnosed during pregnancy (154), affects up to 20% of pregnancies worldwide (155), and it is frequently associated with uteroplacental insufficiency, as well as increased risk of IUGR and PE (156, 157).

Morphological and structural abnormalities have been documented in human GDM placentas (158, 159) as well as in placentas from hyperglycemic animal model, resulting in IUGR (160). Placentas from hyperglycemic-induced animal model exhibited disrupted trophoblast invasion, inadequate spiral UA remodeling (160), as well as dysregulation in PGI_2_ levels (161, 162) and decreased levels of VEGF and PlGF (163, 164).

Collectively, the molecular, structural and functional abnormalities observed in GDM pregnancy suggest a defective vascularization due to impaired placental development in GDM pregnancy, affecting the uteroplacental circulation and thus compromising the UPBF.

### 3.4 Placenta accreta spectrum

PAS encompasses a spectrum of conditions characterized by abnormal placental adhesion to the uterine wall, which fails to detach at birth (165). The vast majority of PAS cases are attributed to scarring from cesarean births, which disrupts the endometrial-myometrial interface and promotes aberrant implantation (166–168). In PAS, EVTs excessively invade the myometrium (169), often within a rigid, collagen-rich extracellular matrix (170), accompanied by inflammation and hypoxia in uterus (171, 172). The absence of proper decidualization and EVT invasiveness prevents the remodeling of the uterine spiral artery in PAS (173), resulting in an abnormal pulsatile and high-velocity blood flow at the feto-maternal interface (174). Histopathologic analysis has revealed excessive vascularity in scarred uterine tissue in PAS (173) (175) and a lack of structural integrity of the vessel, potentially due to Von Willebrand factor suppression as demonstrated in ECs of PAS epiplacental artery (176).

The excessive vascularization is supported by upregulation of VEGF, angiopoietin-2 (177) and PlGF (178) in PAS lysates, and reduced expression of antiangiogenic factors such as vascular endothelial growth factor receptor-2 (VEGFR-2), endothelial cell tyrosine kinase receptor (Tie-2), and sflt-1 in syncytiotrophoblast cells from PAS placenta specimens (177). While evidence on VEGF levels in PAS remains controversial (178), hormonal regulation of angiogenic signaling may contribute to this imbalance. Indeed, the relaxin gene is upregulated in the basal plate of PAS placentas, together with RFXP1 in both the basal plate and villous trophoblast (179).

Animal models have recapitulated this condition, including placental dysplasia, incomplete remodeling of the spiral arteries, deep trophoblast invasion at the feto-maternal interface and reduced placental perfusion, as well as imbalances in angiogenic and anti-angiogenic factors within the placenta and in peripheral blood (180).

## 4 Therapeutic interventions

Understanding the molecular mechanisms by which placental hormones and proteins regulate UA adaptations and functions during pregnancy offers novel opportunities for therapeutic strategies in the context of pregnancy diseases. The administration of pregnancy-related hormones and proteins involved in uterine vascular adaptations during healthy gestation may restore the maternal vascular homeostasis in pathological conditions. Remarkably, as naturally occurring molecules synthesized during pregnancy, their administration is less likely to cause adverse effects on maternal and fetal health during pregnancy. As previously discussed, hormones and metabolites secreted by the placenta exert pleiotropic actions during pregnancy, promoting the uterine vascular adaptations to pregnancy. Thus, the preclinical evidence makes them particularly attractive candidates for therapeutic use.

For example, relaxin infusion in women at >40 weeks of gestation did not induce adverse side effects and demonstrated a modest reduction in systolic blood pressure (181). However, relaxin research has advanced to clinical trials primarily in the context of acute heart failure patients, demonstrating good tolerability, improved renal function, and enhanced systemic perfusion following its infusion (182, 183).

Progesterone is another placental hormone with emerging therapeutic potential. Evidence for progesterone beneficial effects during pregnancy arises from the PROMISE trial (184) and the PRISM trial (185), in which first trimester initiation of vaginal progesterone prevented pregnancy loss, lowering the risk of PE and the risk of pregnancy hypertensive disorders (186). Similarly, hCG administration induces a significant improvement in the pregnancy success rate in women with oligomenorrhea (187). Although there were no adverse effects of administering hCG during pregnancy, the evidence supporting its supplementation to prevent recurrent miscarriage remains equivocal (188).

Hence, hormones and proteins secreted by the placenta are naturally occurring molecules that normally circulate in the maternal bloodstream during pregnancy. Although the number of pregnancy-specific clinical trials remains limited, the existing evidence from preclinical and clinical studies indicates a favorable safety profile and mechanistic plausibility for their use during pregnancy. Thus, their administration to women with complicated pregnancy would be expected to ameliorate the maternal vascular dysfunction observed in some pregnancy-related disorders, enabling the activation of several beneficial pathways through the administration of one therapeutic. However, future clinical trials specifically designed to evaluate efficacy, timing of administration, and optimal dosing in women with pregnancy diseases characterized by placental dysfunction will be crucial for improving our understanding of innovative therapeutic interventions.

### 4.1 Placental-target drug delivery systems

The ability of most drugs to reach therapeutic concentrations is limited by the placenta, which acts as a selective barrier between fetal and maternal circulation. To face these challenges, a range of methods for placental-targeted drug delivery have been developed. These include stem cell-based therapies, lipid nanoparticles therapies, and gene therapies.

#### 4.1.1 Stem cell-based therapies

Stem cells are undifferentiated cells, capable of self-renewal and of differentiating into several specialized cells (189). These cells have been isolated from embryonic tissues, placenta, and amniotic fluid (190). Stem cell-based therapies using mesenchymal stromal cells have been shown to hold potential in promoting vascular health (191). *In vitro*, transplantation of mesenchymal stem cells genetically modified to express the heme-oxygenase 1 gene promoted placental vascularization and restored angiogenic balance (192). Accordingly, in a PE-like animal model, transplantation of mesenchymal stem cells modified to express the heme-oxygenase 1 gene alleviates PE symptoms, promoting angiogenesis and improving placental perfusion (193). More recently, exosomes derived from human umbilical cord mesenchymal stem cells have been shown to induce similar therapeutic effects as mesenchymal cells themselves. For instance, in animal models of PE, exosome-derived mesenchymal cells decreased blood pressure and proteinuria (194–196), and decreased the death rate of the fetuses (195) as well as the fetal birth weight (196). Furthermore, human umbilical cord mesenchymal stem cells-derived exosomes have been shown to rescue sFlt-1-induced HUVECs dysfunction *in vitro* (196).

#### 4.1.2 Lipid nanoparticles therapies

The lipid nanoparticles (LNPs) are nanostructured lipid carriers that encapsulate nucleic acids for efficient intracellular delivery (197). LNPs can be classified into liposomes, solid lipid nanoparticles and nanostructured lipid carriers. Drug-loaded LNPs have been successfully employed in the treatment of both acute and chronic disorders (198). However, the use of LNPs-based therapies during pregnancy requires careful consideration, as it introduces unique challenges to drug delivery. A recent study demonstrates that the structural composition and delivery route of LNPs during pregnancy critically influence mRNA delivery efficiency, maternal immune activation and fetal outcomes (199). Thus, there is a need to design pregnancy-adapted LNPs for safe and effective placental-targeted therapies. For example, LNPs encapsulating VEGF-mRNA triggered vasodilation in the placentas of pregnant mice (200), resolved maternal hypertension and partially restored placental vasculature, the local and systemic immune response, and serum levels of sFlt-1 (201). Similarly, LNPs delivering PlGF mRNA to the placenta in pregnant mice achieved efficient protein expression without maternal or fetal toxicity, further supporting their potential therapeutic action in treating placental-related dysfunction (202).

#### 4.1.3 Gene therapy

Gene therapies typically require a vector to introduce gene material into target cells. The vector can be either viral or non-viral, and both have proven effective for transporting therapeutic agents directly to specific organs and cells, including the placenta. Major viral vectors used for gene therapies are adenovirus, lentivirus and adeno-associated virus, which differ in efficiency, duration of gene expression and immunogenicity. For example, in pregnant mice, the use of RGD fiber-mutant adenoviral vectors enhanced placental tropism and gene transfer efficiency by 10- to 100-fold compared to conventional vectors and sustained transgene expression for at least 7 days (203). Notably, RGD fiber-mutant adenovirus vectors did not induce placental dysfunction or fetal loss (203), suggesting a favorable safety profile for RGD fiber-mutant adenoviral vectors during pregnancy. Intra-arterial administration of adenoviral vectors encoding the VEGF gene has been associated with enhanced fetal growth (204, 205) and increased UPBF (52) in animal models.

Among non-viral vectors, nanostructured delivery systems complexed with the IGF-1 gene have shown promising results in pregnancy models. *In vivo* studies in pregnant mice demonstrated that this vector effectively delivered the gene in the placenta and alleviated IUGR (206). Similarly, in guinea pigs, IGF-1 gene delivery promoted vascular remodeling, enhancing the expression of growth factors (207) and increasing the fetal capillary volume density along with fetal glucose transport (208). Accordingly, in ex vivo human placental explants, perfusion with the IGF-1 vector increased human IGF1 expression in villous fragments and enhanced translocation of glucose transporters (209).

In addition to gene transfection approaches, gene silencing strategies using small interfering RNAs represent other possibilities in the treatment of pregnancy-related disorders. In this regard, the use of siRNA for sFlt-1 has been shown to lower maternal blood pressure and to reduce proteinuria in an animal model of PE (210, 211). Thus, comprehensively, these approaches may have the potential to improve the hemodynamic disturbance observed in pregnancy complications such as PE and IUGR.

## 5 Predictive biomarkers and diagnostics

A wide range of serum biomarkers have been investigated as potential markers for early screening of pregnancy complications (see **Tables 2**–**5**).

**Table 2:** Biomarkers associated with PE categorized by trimester of pregnancy at screening.

Biomarker (units)	Type	GW (at sampling)	Control mean	PE mean (phenotype)	Reference
First trimester					
PAPP-A (MoM)	protein	–	0.53	<0.53	(212)
PAPP-A (MoM)	protein	11-13	1.00	0.84	(242)
PAPP-A (ng/mL)	protein	12-14	129	121(mild) 71.7 (severe)	(228)
β-hCG (MoM)	glycoprotein hormone	11-13	1.00	0.92	(242)
PP13 (MoM)	protein	8-13	1.00	0.89 (mild) 0.65 (severe)	(223)
PP13 (MoM)	protein	6-10	–	0.28	(252)
PP13 (pg/mL)	protein	9-12	132.5	27.2	(251)
PP13 (pg/mL)	protein	9-12	225.3 ± 67.3	157.9 ± 45.5	(224)
PP13 (pg/mL)	protein	10-13	54.8	35.8 (severe)	(227)
Inhibin A (pg/mL)	protein	12-14	277	341(mild) 378 (severe)	(228)
Inhibin A (pg/mL)	protein	11-13	231.13	286.64	(242)
activin A (ng/mL)	protein	11-13	2.16	2.52	(242)
Second trimester					
PAPP-A (ng/mL)	protein	18-20	621	661(mild) 596 (severe)	(228)
β-hCG (IU/mL)	glycoprotein hormone	16-18	19165.03 ± 8044.7	27,519.61 ± 7,483.04 (mild) 36,420.27 ± 6,703.07 (severe)	(217)
β-hCG (MoM)	glycoprotein hormone	22-24	0.923	0.933	(216)
Inhibin A (pg/mL)	protein	18-20	195	213 (mild) 340 (severe)	(228)
Inhibin A (MoM)	protein	16-20	<1.25	>1.25	(229)
Inhibin A (MoM)	protein	15-22	0.94	1.45	(230)
Relaxin 2 (MoM)	hormone	15-22	1.019	1.116	(230)
Leptin (ng/mL)	hormone	9-26	20.9	30.5	(236)
Endoglin (MoM)	membrane glycoprotein	15-22	1.00	1.278	(230)
PlGF (pg/mL)	growth factor	15-19	122.4 ± 81	61.3 ± 28.1	(245)
PlGF (pg/mL)	growth factor	14-23	146	86	(246)
sFlt-1 (pg/mL)	protein	14-23	2,353	3,861	(246)
PlGF/sFlt-1	growth factor/protein ratio	14-23	1.2	1.6	(246)
VEGF (pg/mL)	growth factor	15-19	6.03 ± 4.64	2.57 ± 1.45	(245)
Third trimester					
PAPP-A (ng/mL)	protein	26-28	1392	1276 (mild) 1170 (severe)	(228)
PP13 pg/mL	protein	>24	964	1598	(226)
Inhibin A (pg/mL)	protein	26-28	346	430 (mild) 1250 (severe)	(228)
Relaxin (ng/mL)	hormone	20-41	0.42 ± 0.05	0.24 ± 0.03 (mild) 0.23 ± 0.01 (severe)	(232)
PTHrP (pg/mL)	protein	21-28	452.7 ± 9.04	355 ± 3.38	(233)
Leptin (ng/mL)	hormone	37-39	10.8 ± 5.3	26.6 ± 10.9	(237)
CRH (pmol/l)	hormone	31-35 36-38	195 ± 44 669 ± 87	1020 ± 304 1390 ± 257	(239)
NPY (pmol/m)	neuropeptide	–	23.20	40.90	(240)
NPY (ng/mL)	neuropeptide	37-42)	0.5 ± 0.02	0.5 ± 0.03	(117)
NPY2R (gene expression)	neuropeptide receptor	37-42	4.5 ± 1.375	1.2± 0.440	(117)
activin A (mIU/L)	protein	8-38	4.34	not different	(241)
Igf-1 (ng/mL)	growth factor	29-42	113.5 ± 16.6	87.7 ± 17.5	(243)
PlGF (log)	growth factor	30-33	3.7 ± 1.6	2.9 ± 1.4	(244)
sFlt-1 (pg/mL)	protein	26-40	1731.5	9581	(247)
sFlt-1/PlGF	protein/growth factor ratio	26-40	3.96	158	(247)

GW, gestational week.

**Table 3:** Biomarkers associated with IUGR categorized by trimester of pregnancy at screening.

Biomarker (units)	Type	GW (at sampling)	Control mean	IUGR mean	Reference
First trimester					
PAPP-A (MoM)	protein	11-14	-	≤0.4	(248)
PAPP-A (difference in median)	protein	11-14	1	0.64	(295)
PAPP-A (MoM)	protein	11-13	1.15 ± 0.59	0.79 ± 0.38	(249)
β-hCG (MoM)	glycoprotein hormone	11-13	1.36 ± 0.85	1.52 ± 0.94	(249)
PP13 (pg/mL)	protein	9-12	132.5	86.6	(251)
PP13 (MoM)	protein	6-10	-	0.44	(252)
Third trimester					
Leptin (ng/mL)	hormone	33-41	37.17 ± 28.07	52.73 ± 30.49	(255)
CRH (pg/mL)	hormone	33	137.0 ± 11.3	232.0 ± 43.5	(256)
CRH (fold change)	hormone	29-34	-	40	(257)
PAPP-A (fold change)	protein	29-34	-	5	(257)
Inhibin A (pg/mL)	protein	20-40	0.55 ± 0.21	0.76 ± 0.27	(258)
PSG-1 (fold change)	protein	29-34	-	5	(259)
PlGF (MoM)	growth factor	26-31	0.43	0.26	(260)
PlGF (median)	growth factor	35-40	13.8	12.14	(264)
sFlt-1 (MoM)	protein	26-31	1.74	4.62	(260)
sFlt-1/PlGF (MoM)	protein/growth factor ratio	26-31	5.88	38.76	(260)
sEng (ng/ml)	protein	20-37	5.3	25.9	(263)
sEng (ng/ml)	protein	36-39	10.6 ± 3.7	12.2 ± 4.3	(266)
VEGF (median)	growth factor	35-40	12.98	10.81	(264)
Flt-1 (median)	protein	35-40	15.5	14.9	(264)

GW, gestational week.

**Table 4:** Biomarkers associated with GDM categorized by trimester of pregnancy at screening.

Biomarker (units)	Type	GW (at sampling)	Control mean	GDM mean	Reference
First trimester					
Relaxin 2 (ng/mL)	hormone	12	0.47	0.83	(268)
PAPP-A (MoM)	protein	<14	0.99	0.97	(273)
PAPP-A (MoM)	protein	11-13	1.2 ± 0.7	0.8 ± 0.5	(274)
β-HCG (MoM)	glycoprotein hormone	<14	1.02	1.05	(273)
β-HCG (MoM)	glycoprotein hormone	11-13	1.2 ± 0.5	1 ± 0.5	(274)
PlGF (log10)	growth factor	11-14	1.68	1.76	(280)
PlGF (MoM)	growth factor	8-14	1.03 ± 0.48	1.05 ± 0.38	(281)
Second trimester					
β-HCG (MoM)	glycoprotein hormone	12	1.03 ± 0.50	0.96 ± 0.48	(272)
PAPP-A (MoM)	protein	12	1.09 ± 0.50	1.00 ± 0.51	(272)
PP13 (MoM)	protein	16-20	1.16	1.47	(275)
sFlt-1 (MoM)	protein	16-20	1.04	0.66	(275)
VEGF-A (pg/mL)	growth factor	23-29	146.60 ± 12.03	296.92 ± 17.37	(265)
Endoglin (pg/mL)	protein	23-29	1444.78 ± 82.30	1814.06 ± 141.10	(265)
Third trimester					
Relaxin 2 (pg/mL)	hormone	-	439	667.5	(269)
Leptin (ng/mL)	hormone	28	18.2 ± 1.5	24.9 ± 1.6	(277)
Leptin (ng/mL)	hormone	24-28	24.10	30.60	(278)

GW, gestational week.

**Table 5:** Biomarkers associated with PAS categorized by trimester of pregnancy at screening.

Biomarker (units)	Type	GW (at sampling)	Control mean	PAS mean	Reference
First trimester					
PAPP-A (MoM)	protein	-	0.89	1.96	(284)
PAPP-A (IU/L)	protein	11-13	5.34	3.63	(286)
β-hCG (IU/L)	glycoprotein hormone	11-13	33.5	51.4	(286)
Second trimester					
alpha-fetoprotein (MoM)	protein	16-19	1.08	1.17	(290)
alpha-fetoprotein (MoM)	protein	-	0.98 ± 0.60	1.49 ± 0.54	(291)
β-hCG (MoM)	glycoprotein hormone	16-19	1.02	1.36	(290)
Third trimester					
PL (copies/mL)	hormone	28-32	90	615	(288)
PL (MoM)	hormone	-	1.00	2.78	(289)
β-hCG μg/L	glycoprotein hormone	26-34	11.8 ± 8.8	7.8 ± 5.9	(287)
sFlt-1 (pg/mL)	protein	33-39	25,779.2	9407.1	(292)

GW, gestational week.

### 5.1 Biomarkers for diagnosing PE

#### 5.1.1 Hormonal and protein biomarkers

PAPP-A levels <10^th^ percentile in the first trimester are associated with an increased risk of PE (209, 212). Its predictive value improves when combined with other markers, such as β-hCG (213–215) and UA pulsatility index (216).

In the early second trimester of pregnancy (16–18 weeks of gestation), elevated β-hCG levels have been associated with PE and particularly severe PE (217). However, other studies demonstrate inconsistent findings (216, 218). A recent meta-analysis confirmed increased levels of serum β-hCG in the early second trimester, but not during the first trimester, in pregnancies later complicated by PE compared to healthy pregnancies (219).

Another biomarker used to predict PE is A-Disintegrin and Metalloprotease-12 (ADAM-12) decreased levels, in association with PAPP-A, correlate with the severity of IUGR (220), though findings are inconsistent for ADAM-12 association with PE and related disorders (221). Conflicting evidence, such as increased ADAM-12 levels reported in PE and HELLP syndrome (222), underscores the need for further validation.

Research has also been conducted on the use of PP13 in the prediction of PE. Low first-trimester levels are associated with PE risk (223–225), while increased amounts of PP13 carried via the placental-associated extracellular vesicles have been reported in late gestation (226). Combined with PAPP-A and the free leptin index, PP13 can reach a detection rate of 40% at a 10% false positive rate (227). Inhibin A levels are elevated during both the first trimester (228) and the second trimester (229) in pregnancies complicated by PE, and its levels correlate with the incidence and severity of PE (229). However, to increase its predictive value, the level of inhibin-A is used in combination with other biomarkers such as PAPP-A (228), endoglin and PlGF (230). Additional hormones have also been explored for PE screening. For example, relaxin levels decrease during the first trimester of pregnancy and have been associated with late-onset PE (231), though relaxin levels may not reflect disease severity (232).

Similarly, decreased serum levels of PTHrP (233) E2 and progesterone (234) are lower in preeclamptic pregnancies compared to healthy pregnancies.

A reduction in PL levels has also been associated with PE onset, making it a reliable marker of placental function in the second half of pregnancy (235).

Conversely, leptin concentration is significantly increased in PE, both early and late in gestation (236, 237).

CRH levels increase in late pregnancy in women with PE (238, 239), with a sensitivity of 92.5% and specificity of 82.5% as a single predictive marker for PE (238).

Elevated serum NPY levels have also been reported in PE during the third trimester (240). Interestingly, another study found a decrease in NPY2 receptor rather than NPY expression in late pregnancy (117).

Finally, the role of activin A remains unclear. Although no significant difference between PE and healthy pregnancy has been demonstrated in its levels through gestation (241), a slight increase in the first trimester was observed in another study; however, the association remains too weak to support its use in PE screening (242).

#### 5.1.2 Growth factors biomarkers

A recent study found that the levels of IGF-1 and its receptor in the serum and in placental tissue were significantly lower in the preeclamptic group compared to a control group. IGF-1 exhibited a sensitivity of 86% and a specificity of 100% for diagnosing PE, while serum IGF-1R showed a sensitivity and a specificity of 77% for diagnosing PE (243). Angiogenic factors have emerged as a useful tool for the identification of patients at risk for pregnancy complications. The lowering of PlGF alone (244) or in association with VEGF (245), as well as in association with sFlt-1 (246), has been associated with PE. In addition, increased sFlt-1, sFlt-1/PlGF ratio, and a decline in PlGF levels have also been associated with PE (247). Further, PlGF in association with PAPP-A may predict early onset PE during the third trimester (228).

### 5.2 Biomarkers for diagnosing IUGR

Hormonal and protein biomarkers - Low levels of PAPP-A during the first trimester have been consistently associated with IUGR (248, 249). The predictive value improves when PAPP-A is combined with UA Doppler pulsatility index (249).

While some studies found no significant association between β-hCG and the occurrence of IUGR (249), a meta-analysis reported increased levels of β-hCG during the second trimester in pregnancy complicated by IUGR (250).

PP13 has shown potential as a predictive biomarker for IUGR, particularly when assessed during the second trimester, where PP13 levels are significantly. However, its predictive accuracy is higher for PE than for IUGR (251). Nonetheless, maternal serum PP13 in the first trimester may predict the risk of developing IUGR, PE, or PE complicated by IUGR (252). However, PP13 role in predicting IUGR remains contradictory (253).

A positive correlation between maternal PL levels and estimated fetal weight has been reported in a cross-sectional study, suggesting its potential utility in monitoring fetal growth (235, 254). Importantly, PL reduction has been significantly associated not only with the onset of PE, but also with asymmetrical IUGR, supporting its role as a marker of placental dysfunction (235).

Elevated maternal serum leptin concentrations at delivery have been documented in pregnancies complicated by IUGR compared to normal pregnancies (255). Similarly, elevated CRH levels in the third trimester of pregnancy have been associated with a 3.6-fold increase in IUGR risk (256). Molecular studies using in silico approaches further support these findings, showing increased RNA expression of PAPP-A2 and CRH in both maternal blood and placental tissue from IUGR pregnancies (257).

Additionally, higher levels of inhibin A and B have been observed in fetuses affected by IUGR compared to controls (258). Lastly, lower levels of serum PSG-1 have been reported in the late second trimester of IUGR pregnancies (259).

Growth factors biomarkers **–** During the third trimester, pregnancies with IUGR showed significantly lower levels of free and total PlGF (260). A meta-analysis revealed that pregnant women with IUGR and PE may have higher sFlt-1/PlGF ratio. A sFlt-1/PlGF ratio >33 showed strong predictive capacity in FGR (261). Nonetheless, similar to Flt-1/PlGF, sEng has high predictive value for IUGR along with PE and HELLP (262). IUGR pregnancies, along with PE, showed significantly elevated sEng concentrations and a strong positive correlation with sFlt-1 (263). Remarkably, placental transcript abundance for VEGF was significantly lower in IUGR placentae compared to healthy placentae (264). Additionally, increased sEng levels have been consistently associated with GDM, PE, and IUGR across different stages of gestation (265, 266). Despite these associations, a meta-analysis by Conde-Agudelo et al., including 53 studies and 39,974 women, concluded a minimal predictive accuracy of angiogenic factors for IUGR (267).

### 5.3 Biomarkers for diagnosing GDM

#### 5.3.1 Hormonal and protein biomarkers

Plasma levels of relaxin-2 have been found to increase in both the first- (268) and third-trimester of pregnancy in women with GDM (269).

The association between *β*-HCG and GDM appears more controversial. One study reported that the incidence of GDM was increased in women with elevated *β*-HCG levels during the second trimester of pregnancy (250, 270). In contrast, Liu et al., found that increased *β*-HCG levels in early pregnancy were associated with a lower risk of GDM (271), raising the possibility of a time course-dependent profile in the predictive capacity. Supporting this complexity, a machine learning study demonstrated improved prediction accuracy for GDM when *β*-HCG was used in combination with PAPP-A (272).

Although PAPP-A alone may not be a definitive predictive marker for GDM (273, 274) low levels could support the recommendation for early screening as part of a broader diagnostic approach. Low serum PAPP-A MoM levels are significantly associated with the development of GDM,

Increased serum levels of PP13 in the early second trimester were significantly associated with GDM, with a detection rate of 92.3% at 80% specificity (275). This preliminary study may support PP13 as an early screening biomarker, though its implementation in clinical practice requires validation.

PL has also been implicated in GDM. A meta-analysis showed that in women with type 1 GDM, PL is decreased in early pregnancy and increased in late pregnancy (276), suggesting that PL dynamics may reflect placental adaptation to altered glycemic states.

In addition, increased leptin levels were observed in the third trimester of pregnancies complicated by GDM compared to normoglycemic pregnancies (277, 278). Accordingly, a meta-analysis confirmed these data, revealing increased leptin concentrations in GDM cases regardless of gestational age, with no trimester-dependent variation in its level (279).

#### 5.3.2 Growth factors biomarkers

Similar, GDM has been associated with increased levels of sFlt-1 (280). However, the predictive value of PlGF in GDM remains uncertain (244, 281). Accordingly, increased levels of VEGF-A, endoglin, endothelin-1, and angiopoietin-2 have also been documented in GDM placentas compared to controls (265). In a longitudinal study, the sFlt-1 levels were higher in the first trimester in GDM women as compared to non-GDM women. Placental PlGF and Flt-1 levels were lower in the GDM group, and they were negatively associated with placental dimensions, revealing that an imbalance in these growth factors may affect placental development, hence pregnancy outcomes in GDM (282). Additionally, increased sEng levels have been consistently associated with GDM (283).

### 5.4 Biomarkers for diagnosing PAS

Hormonal and protein biomarkers **-** Increased levels of PAPP-A during the first trimester of pregnancy have been observed in women at higher risk of PAS (284, 285). In contrast, another study reported that PAPP-A levels were lower in PAS cases, while β-hCG levels were increased compared to the control group. Interestingly, combining these biomarkers in predictive models substantially improved diagnostic accuracy over single-marker use, with a sensitivity of 100% and specificity of 72% (286). Studies have also examined the diagnostic value of hyperglycosylated human CG. One study found that levels of this hormone were lower in PAS cases compared to controls in both the second and third trimesters of pregnancy. However, its diagnostic performance was limited, showing only a modest sensitivity and specificity (287).

Another hormone of interest is PL. Research has shown that during the third trimester, PL mRNA levels are increased in the plasma of women with invasive placentation (288). These results are in agreement with the study from Jing et al., which confirmed that maternal plasma PL mRNA concentrations were significantly increased in PAS cases compared to controls (289).

In addition to protein hormones, alpha-fetoprotein (AFP), a glycoprotein produced by the fetal liver and transported to maternal serum through the placenta or by diffusion across fetal membranes, has also emerged as a potential biomarker for PAS. A retrospective case-control study found increased levels of AFP in the PAS group during the second trimester, with a sensitivity of 71% and a specificity of 46% (290). Similar results were obtained in a subsequent study, which demonstrated a positive association between elevated second-trimester serum AFP and placenta accreta, suggesting its potential use as a screening tool for pregnancy at high risk of PAS (291).

Growth factors biomarkers - PAS is characterized by an excessive neovascularization, thus angiogenic factors have also been studied as potential biomarkers. A study found significantly increased maternal serum PlGF levels in PAS cases compared to the healthy group during the third trimester, while no differences were found in serum levels of VEGF (178). In the same study, PlGF is elevated in maternal serum and the placental bed of patients with PAS disorders compared to those without PAS (178).

In contrast, studies investigating sFlt-1 levels have yielded less conclusive results. One study reported no association between third-trimester sFlt-1 levels and PAS disorders (292). Another study suggested that although sFlt-1 and PlGF expression slightly differ depending on the depth of placental invasion, there is no direct correlation between serum levels and PlGF and sFlt-1 expression in the placenta (293).

Although these biomarkers show potential for detecting adverse pregnancy outcomes, large-scale studies are needed to conclude whether they could be used in early screening for pregnancy complications such as IUGR, PE, GDM, and PAS.

## 6 Future research directions

There is still much to learn about how the placental hormones and metabolites contribute to maintaining basal and activity-dependent perfusion of the uterine circulation during pregnancy. Therefore, it is critical for basic science and clinical studies to elucidate the precise molecular mechanisms by which hormones, growth factors, and proteins secreted by the placenta modulate uterine vascular adaptation to pregnancy. A deeper understanding of these pathways is essential to inform clinical strategies for early diagnosis, risk stratification, and targeted therapy. Placental hormones and metabolites represent promising therapeutic targets for the prevention and treatment of pregnancy complications, including PE and IUGR. Integrative approaches combining translational models, omics technologies, and longitudinal clinical studies will be critical to unlocking their potential and improving maternal and fetal outcomes.

## 7 Conclusion

The endocrine function of the placenta is central to the hemodynamic adaptations of the uterine circulation during pregnancy. The placental hormones and metabolites appear to have a substantial effect on the uterine circulation, including altering ECs vasodilator production, decreasing vascular resistance, and promoting the remodeling of the UA. Placental hormones and metabolites also promote the angiogenic process, ultimately contributing to the increase in UPBF necessary to sustain normal fetal growth. Most of these changes occur in the first trimester of pregnancy, a crucial period where abnormalities in the secretion of placental hormones and metabolites may reflect early signs of placental and uterine vascular impairment. Understanding how the secretion of placental hormones and metabolites is altered during gestation, both under normal and pathological conditions, is important for the treatment and prevention of pregnancy complications.
